# CUT&RUNTools 2.0: a pipeline for single-cell and bulk-level CUT&RUN and CUT&Tag data analysis

**DOI:** 10.1093/bioinformatics/btab507

**Published:** 2021-07-09

**Authors:** Fulong Yu, Vijay G Sankaran, Guo-Cheng Yuan

**Affiliations:** Department of Pediatric Oncology, Dana-Farber Cancer Institute, Boston, MA 02215, USA; Division of Hematology/Oncology, Boston Children’s Hospital, Boston, MA 02115, USA; Department of Pediatrics, Harvard Medical School, Boston, MA 02115, USA; Program in Medical & Population Genetics, Broad Institute of MIT and Harvard, Cambridge, MA 02115, USA; Department of Pediatric Oncology, Dana-Farber Cancer Institute, Boston, MA 02215, USA; Division of Hematology/Oncology, Boston Children’s Hospital, Boston, MA 02115, USA; Department of Pediatrics, Harvard Medical School, Boston, MA 02115, USA; Program in Medical & Population Genetics, Broad Institute of MIT and Harvard, Cambridge, MA 02115, USA; Department of Pediatric Oncology, Dana-Farber Cancer Institute, Boston, MA 02215, USA; Division of Hematology/Oncology, Boston Children’s Hospital, Boston, MA 02115, USA; Department of Pediatrics, Harvard Medical School, Boston, MA 02115, USA; Department of Genetics and Genomic Sciences, Charles Bronfman Institute for Personalized Medicine, Icahn School of Medicine at Mount Sinai, New York, NY 10029, USA

## Abstract

**Motivation:**

Genome-wide profiling of transcription factor binding and chromatin states is a widely-used approach for mechanistic understanding of gene regulation. Recent technology development has enabled such profiling at single-cell resolution. However, an end-to-end computational pipeline for analyzing such data is still lacking.

**Results:**

Here, we have developed a flexible pipeline for analysis and visualization of single-cell CUT&Tag and CUT&RUN data, which provides functions for sequence alignment, quality control, dimensionality reduction, cell clustering, data aggregation and visualization. Furthermore, it is also seamlessly integrated with the functions in original CUT&RUNTools for population-level analyses. As such, this provides a valuable toolbox for the community.

**Availability and implementation:**

https://github.com/fl-yu/CUT-RUNTools-2.0.

**Supplementary information:**

Supplementary data are available at *Bioinformatics* online.

## 1 Introduction

Genome-wide analysis of transcription factor binding sites and chromatin states is essential for understanding cell-type specific transcriptional regulatory mechanisms. Recently, a new generation of technologies has emerged with enhanced sensitivity and efficiency (Ai *et al.*, 2019; Carter *et al.*, 2019; Hainer *et al.*, 2019; Kaya-Okur *et al.*, 2019; Skene and Henikoff, 2017). As a result, it has become possible to profile genome-wide occupancy analysis in a limited number of or even single cells. In previous work, we developed CUT&RUNTools for analyzing CUT&RUN data, providing an end-to-end CUT&RUN data analysis pipeline that includes sequence alignment and pre-processing, peak calling, cut matrix estimation, motif and footprinting analyses and additional analyses (Zhu *et al.*, 2019). Here, we have further extended this software by implementing a flexible pipeline for single-cell data quality assessment, analysis and visualization, thus enabling users to rapidly utilize new technologies to systematically dissect the heterogeneity of the epigenomic landscape and gene regulatory networks among individual cells. In addition, we have also implemented a number of new features, including data normalization, peak calling and downstream functional analysis that improve the performance for bulk data analysis.

## 2 Description

**Fig. 1.**
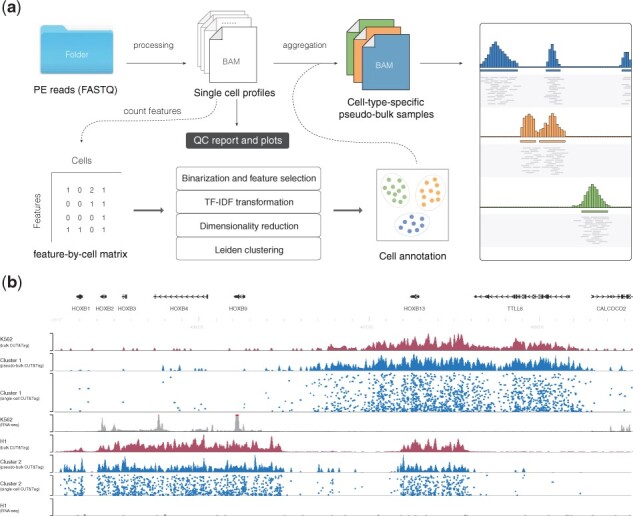
Overview of CUT&RUNTools 2.0. (**a**) The workflow of single-cell data processing and analysis and (**b**) the genome browser tracks for the HOXB gene locus

CUT&RUNTools 2.0 provides a new module to facilitate the analysis and visualization of single-cell resolution data. The module implements a flexible, end-to-end pipeline that takes raw data as input, followed by a number of steps including data preprocessing and quality assessment, feature extraction, dimensionality reduction, cell clustering, data aggregation and visualization. A number of computational methods have been developed for single-cell ATACseq analysis (Baker *et al.*, 2019; Bravo Gonzalez-Blas *et al.*, 2019; Granja *et al.*, 2021; Ji *et al.*, 2017, 2020; Pliner *et al.*, 2018; Schep *et al.*, 2017; Urrutia *et al.*, 2019; Xiong *et al.*, 2019; Zamanighomi *et al.*, 2018), and the performance of these methods has been systematically benchmarked (Chen *et al.*, 2019). CUT&RUNTools 2.0 builds on a number of existing tools. In addition to those already included in the original version, we also implemented a number of additional tools including GNU parallel, umap-learn and several other scripts (see Supplementary Material for more details). CUT&RUNTools 2.0 also adds a number of new features for single-cell analysis to enhance scalability and usability, including (i) supporting multiple input options including raw FASTQ files and reads alignment and processing in parallel; (ii) three complementary options for feature selection and (iii) generating customized genome-browser tracks to facilitate informative and time-efficient data visualization. Importantly, CUT&RUNTools 2.0 provides a convenient platform to combine single-cell and bulk data analysis in a single software package so that the utilities in the original CUT&RUNTools can be easily accessed. An overview of the single-cell pipeline is shown in Figure 1a.

The input FASTQ files are processed by read trimming, mapping and filtering. The trimmed reads are aligned to human/mouse reference genome. For each cell, only high mapping quality, uniquely aligned and properly mapped reads are retained for further analysis. CUT&RUNTools 2.0 reports a set of common quality control (QC) metrics as a summary report and diagnostic plots, which can be conveniently used for the data quality evaluation. In addition, single-cell level QC measures are saved and can be used to filter out low-quality cells based on user-customized criteria.

Due to the sparsity of single-cell data, sequence reads falling into a set of pre-selected features are aggregated. CUT&RUNTools 2.0 provides three options for feature selection: peaks from cell aggregation, genome-wide bins and user-defined functional elements. In each case, a feature-by-cell matrix is derived by counting the sequence reads that fall into a pre-identified feature across individual single cells in parallel. Furthermore, the count matrix is binarized to reduce noise associated with low-number counts.

To reduce dimension, the resulting feature-by-cell matrix is processed by singular value decomposition, which generates a Latent Semantic Indexing (LSI) score matrix (Cusanovich *et al.*, 2015) to further perform the dimensionality reduction and clustering analysis. The cells from the same cluster are merged into a pseudo-bulk profile and the corresponding genome track files for both individual cells and the pooled signal are automatically generated per cell population. These pseudo-bulk samples are compared by analysis of distinct peaks, motif discovery, footprints or functional enrichment. The main processing steps in the data processing and feature-by-cell matrix construction can be performed in parallel to make full use of the available computational resources and reduce runtime. Users can either run the entire workflow or select a specific step by customizing the configuration file (Details of these functions are provided in the Supplementary Material).

## 3 Results

To demonstrate its utility, we applied the CUT&RUNTools 2.0 pipeline to re-analyze a publicly available single-cell CUT&Tag (scCUT&Tag) dataset (Kaya-Okur *et al.*, 2019). In this study, the investigators profiled genome-wide occupancy of H3K27me3, a repressive histone mark, in individual cells from two distinct cell lines: H1 (human embryonic stem cells) and K562 (a human erythroleukemia cell line). It takes approximately 3 h to finish the entire analysis pipeline using a MacBook computer with 8 cores and 16 GB of RAM.

A summary report regarding a set of QC metrics and the corresponding diagnostic plots for the experiment were produced (Supplementary Fig. S1). Overall, a total of 1373 cells were detected and approximately 0.14 million reads per cell were sequenced. For most cells, more than 99% of the reads were successfully mapped to the reference sequence indicating a high degree of purification. We also found that a vast majority of cells having a high proportion (median percentage, 99.5%) of nuclear reads (reads not aligned to mitochondrial DNA) in each single-cell library. Less than 1% of duplicated reads were found for the majority of cells, suggesting the libraries of individual cells were sequenced near saturation. The fragment size was calculated as the length between the cut point of the Tn5 enzyme and the average size is 230.3 bp, which is expected for typical histone modification and longer than typical transcription factor binding profiles (∼120 bp) (Kaya-Okur *et al.*, 2019; Skene *et al.*, 2018; Zhu *et al.*, 2019). The fragment size distribution of all the reads from individual cells exhibits a clear nucleosomal binding pattern. These quality metrics were reported as a summary table (Supplementary Fig. S1a) as well as a number of diagnostic plots (Supplementary Fig. S1b and c). The high quality of the data is reflected by a number of factors including high alignment ratio, the ideal proportion of properly mapped reads, high-quality mapping reads and nuclear reads and a high level of library complexity.

Next, we aggregated sequence reads from individual cells into a pooled sample, and then applied MACS2 (Zhang *et al.*, 2008) to detect peaks. In order to preserve the structure of the data, we used a permissive cutoff of *q*-value < 0.01, which detects a total of 379 566 peaks. We assessed the signal-to-noise ratio in individual cells based on the fraction of reads that fall into the detected peaks. Overall, the signal-to-noise ratio ranges from 28% to 68%, with a median level of 45%. Of the 1373 cells, three did not pass the QC criteria because they were associated with either a low signal-to-noise ratio (<30%) or a small number of qualified fragments (<10 000), therefore these three cells were excluded from further analysis (Supplementary Fig. S1d).

For the remaining 1370 cells, we created a binarized feature-by-cell matrix indicating the presence or absence of a peak of any individual cell. We also removed features that were either ubiquitous (detected in > 80% cells) or rare (detected in < 0.1% cells) therefore unlikely to be informative. After dimensionality reduction and clustering, two distinct cell populations were identified (Supplementary Fig. S2a), which matched nearly perfectly to the true cell-type labels (Supplementary Fig. S2b): all the cells in cluster 1 were K562 cells, whereas nearly all the cells in cluster 2 were H1 cells, indicating the biological information was preserved by our single-cell CUT&Tag analysis pipeline.

To compare the genome-wide H3K27me3 profiles for different cell clusters, the reads obtained from all the cells in each cluster were aggregated to create a pseudo-bulk sample. We further downloaded and processed the cell-type matched bulk data and found the pseudo-bulk samples are highly correlated with the corresponding bulk data (Supplementary Fig. S2c). Together, these results suggest our single single-cell analysis is able to extract useful information and accurately reveal the cellular heterogeneity.

To aid visualization, we created genomic tracks files of not only the pooled signals, but also binding profiles at the single-cell resolution for different cell clusters (Fig. 1b). This visualization clearly shows the differences between the H1 and K562 cells. Of note, H3K27me3 occupies across the entire HOXB cluster in H1 cells, but only partially occupies a broad domain around the HOXB13 locus in K562 cells (Fig. 1b). By comparing with ENCODE RNA-seq data, we found this change of H3K27me3 profiles is consistent with transcriptional activity differences between these two cell types, where HOXB1-9 genes are expressed in K562 cells but the entire HOXB cluster genes are repressed in H1 cells (Fig. 1b).

The pseudo-bulk data were used to further characterize and compare the H3K27me3 landscape between different cell subpopulations. We first identified 75 812 peaks in cluster 1 (corresponding to K562 cells) and 25 064 peaks in cluster 2 (corresponding to H1 cells) by using a stringent cutoff of *q*-value < 0.01 and fold change > 5 (Supplementary Fig. S3a). We found only a small proportion of peaks (1525) overlapping between these two clusters. More peaks were associated with non-coding regions comparing to coding regions in both cell clusters (Supplementary Fig. S3b). Of note, a much larger proportion of peaks of cluster 2 (17%) were proximal to transcriptional start sites compared to cluster 1 (5%), suggesting that more embryonic associated genes may be more directly regulated by repressive H3K27me3 domain. We identified potential regulators closely related to the repression of cell-type-specific genes and cis-elements, such as the tumor suppressor Transcription Factor AP-2 Beta and Early B cell factor 1 in cell cluster 1 (Bohle *et al.*, 2013; Lightfoot *et al.*, 2004) (Supplementary Fig. S3c) and the development associated TF early growth response protein 2 in cell cluster 2 (Du *et al.*, 2014; Wilkinson *et al.*, 1989) (Supplementary Fig. S3d). Gene Ontology analysis showed that many different cell and system development associated functions including embryo development, system development, cell differentiation and multi-cellular organism development were markedly enriched in cluster 2, which also supports that the establishment and removal of H3K27me3 at specific genes in the embryonic stem cells is critically important for normal development.

## 4 Discussion

In response to recent development of single-cell CUT&RUN and CUT&Tag technologies, we have extended our CUT&RUNTools package by adding a single-cell analysis module. This module builds upon existing single-cell ATACseq analysis tools and provides a number of additional features to enhance performance and usability. In addition, CUT&RUNTools 2.0 also contains a number of updates in bulk-level analyses, such as spike-in sequence alignment and data normalization. More importantly, CUT&RUNTools 2.0 seamlessly integrates single-cell and bulk-level analyses in one package, providing the convenience to study multiple datasets in a standardized manner. As single-cell multi-modal data become increasingly available, CUT&RUNTools 2.0 provides as a convenient toolkit facilitating integration which in turn will provide a better understanding of epigenomic heterogeneity and regulatory logic in both healthy and diseased tissues.

Due to the inherent sparsity and high dimensionality of the single-cell epigenome data, appropriate data normalization and dimension reduction are crucial for cell clustering and annotation. A number of methods have been developed for dimensionality reduction. For example, SnapATAC uses a regression-based normalization method to account for differences in library size between cells, and PCA is used to reduce dimensionality before clustering (Fang *et al.*, 2021). chromVAR calculates z-scores to measure gain or loss of accessibility within peaks containing the same motif or annotation (Schep *et al.*, 2017). cisTopic detects cell states and cis-regulatory regions from topic distribution by using latent Dirichlet allocation analysis (Bravo Gonzalez-Blas *et al.*, 2019). LSI normalizes reads using the TF-IDF and reduces dimensionality using SVD on the feature-by-cell matrix (Cusanovich *et al.*, 2015). We have chosen to implement LSI because it was recommended by a previous benchmark analysis (Chen *et al.*, 2019). In future work, we will also implement alternative strategies to enhance robustness and reproducibility.

Our analysis indicates that CUT&RUNTools 2.0 performs well in identifying distinct cell types along with cell-type specific regulatory elements. However, it is important to recognize the dataset we analyzed is highly idealized, where the cell population was artificially created by mixing cells from two well-characterized cell lines. For a real biological dataset, the situation can be much more complex, therefore our performance estimate is likely to be over-optimistic. As new datasets become available, we will re-evaluate the performance of CUT&RUNTools 2.0 to obtain more realistic assessments.

## Supplementary Material

btab507_Supplementary_Data
