# Supplementary material for: CUT&RUNTools 2.0: a pipeline for single-cell and bulk-level CUT&RUN and CUT&Tag data analysis
Source: Bioinformatics. 2021 Jul 9;38(1):252–4. doi: 10.1093/bioinformatics/btab507 (PMC8696090; doi:10.1093/bioinformatics/btab507)
Supplement: btab507_Supplementary_Data [file btab507_supplementary_data.docx]

**CUT&RUNTools 2.0 (Supplementary Information)**

**Materials and Methods**

*Adaptor trimming and reads mapping*

For each cell, the adaptor sequence and primer oligo sequence from the 3’ ends of reads is trimmed off using a two-step trimming strategy consistent with bulk data processing. All the reads are aligned to the corresponding reference genome hg38 using Bowtie2 software (1) with the default parameter. The SAMtools (2) is used to sort and index the resulting BAM files.

*Quality control metrics*

CUT&RUNTools 2.0 provides a set of quality assessment metrics for the overall experiment as well as each barcode cells. The metrics include overall alignment ratio, the number of total reads, properly paired reads, duplicated reads, high-quality reads (MAPQ score >30), and nuclear reads, fragment size, signal-to-noise ratio (the fraction of reads in peaks). The Picard (https://broadinstitute.github.io/picard) *MarkDuplicates* function is used to locate and tag duplicate reads. The unmapped, low quality (MAPQ score <30), unproperly paired and duplicated fragments are discarded, and the remaining data are defined as the qualified fragments. Then, the filtered and sorted BAM files for each cell are generated for further analysis. The fraction of reads in peaks are calculated using BEDTools *genomecov* function (3).

For each run, a summary report is generated by using a custom script, which contains a summarization of the mapping statistics of the overall experiment. Additionally, several diagnostic plots are also produced for intuitive illustration of the quality control metrics across all the cells. We provide two parameters, *num_reads_threshold* (number of unique mapped reads, 10,000 as default) and *percentage_rip* (fraction of reads in peaks, 30% as default) as filters to remove low quality cells. Finally, two files, *statistics_QCpassed.txt* and *statistics_QCfailed.txt,* are generated to record the identity of the filtered cells along with their associated statistics.

*Parallel processing*

Comparing to bulk data, a single-cell CUT&RUN / CUT&Tag experiment usually contains a large number of cells, but the reads in each cell are less abundant. The data processing time roughly scales linearly with the cell number, therefore it may take a long time if the number of cells is large. To overcome this challenge, we have adopted the GNU parallel technique (4) in the main steps of data processing, which results in dramatic reduction of runtime.

*Peak calling*

For the pseudo-bulk data aggregated from cells from the entire datasets or a specific cell cluster, CUT&RUNTools 2.0 enables peak calling by using different methods. By default, we use the MACS 2 narrow peak mode for peak calling (5), which has good performance for TFs (6). In addition, we also implement two alternative strategies: the MACS 2 broad peak mode, and the SEACR algorithm (7). The users can easily make selection by modifying the parameters of *peak_caller* and *peak_thresholds*.

*Construction of feature-by-cell matrix*

CUT&RUNTools 2.0 provides three options to build the feature-by-cell matrix, which can be customized by setting *matrix_type* as *peak-by-cell, bin-by-cell*, *or* *customFeature-by-cell*. *peak-by-cell* refers to using peaks detected from the pooled sample as the feature set, which is selected in the present study. *bin-by-cell* refers to segmenting the genome into equal-size bins (5 kb by default). Using the *customfeature-by-cell* option, users can also upload their own features of interest, such as a list of enhancer regions (input needs to be in the standard BED format).

Once the feature file is designated, CUT&RUNTools 2.0 automatically excludes features overlapping with ENCODE blacklist regions (8) or uninterested chromosomes. For CUT&RUN experiments, an additional filtering step is carried out by removing the regions overlapping with TA repeats regions because these regions usually occur as containment abnormally enriched reads (9). The users can simply set the *experiment_type* parameter as CUT&RUN to turn on this feature. With the preparation of feature files, CUT&RUNTools calculates the read coverage profiles for all the qualified cells, and the resulting feature-by-cell count matrix is generated and saved.

*Count matrix processing*

Owing to the sparsity of count matrix of single-cell epigenomic data, CUT&RUNTools 2.0 converts the feature-by-cell matrix into the sparse Matrix format to allows more efficiency of memory usage and computation by using the ‘Matrix’ package in R (10). The sparse matrix is binarized and an additional filter is used to remove features that are present only in few cells (the maximum of 0.1% of the cells) or in the vast majority of cells (80% of the cells) to efficiently capture the informative signals.

*Dimensionality reduction, cell clustering and visualization*

Dimensionality reduction is performed using the Latent Semantic Indexing (LSI) method, a technique commonly used for document indexing process in natural language processing (11). Based on the resulting LSI matrix, the graph-based Leiden algorithm is used as the unsupervised clustering method which is implemented with the *leidenalg* and *igraph* library (12, 13). Using the top 30 principal components (different numbers can be tuned by changing parameter *cluster_pc*), we build a shared neighbor network (SNN) graph by considering each cell as a node and further finding its k-nearest neighbors according to the Euclidian distance. Another parameter of *cluster_resolution* is also provided, which is important to control the number of resulting clusters, where the larger of this parameter usually lead to the larger number of resulting cell clusters. The predicted cell type labels are then generated. CUT&RUNTools 2.0 visualizes the results by employing three commonly used methods for dimensionality reduction including PCA, UMAP and t-SNE. The corresponding plots of the first two dimensions are generated and saved, respectively.

*Generation of genome browser tracks*

The tracks of pseudo-bulk data are normalized by the counts per million method and generated with bigwig format using the program *bamCoverage* in the deepTools package (14) with 50 bp resolution. The peak files called from pseudo-bulk data are also generated with BED format. For each single cell, the genome-wide read coverage is calculated using BEDTools (3) in parallel with available computational cores. The regions covered by any reads are extracted and a read coverage file is created with BED format. For each cell cluster, the read coverage files of individual cells are combined into a single track with qBED format for convenient visualization (15), with the cells sorted by the number of covered regions in each cell. All the resulting track files could be directly uploaded and visualized using browser apps such as the WashU Epigenome Browser (16).

*Downstream analysis function specific to cell populations*

Analysis of peaks called from pseudo-bulk data of cell clusters is important to uncover the cell-specific gene regulatory elements. The script *peakOverlap* is used to summarize the peak overlap between different cell clusters, the number of common and specific peaks will be identified and visualized as a Venn diagram. The script *eleAnno* is used to annotate the distribution of peaks over different types of genomic features including 5’ UTR, promoter, exon, intron, 3’ UTR, intragenic and intergenic regions. *haystack_motifs* is used for the motif enrichment analysis of peaks to identify cell-type-specific cis-regulatory elements and associated transcription factors (17). *PeakFun* is used for Gene Ontology (GO) analysis of the top 1,000 interested peaks with the search of genes associated with GO ‘biological process’ categories (18) (see the manual on the website for details).

*Newly incorporated tools*

We incorporated a number of software and custom analysis scripts in addition to the original version of CUT&RUNTools.

- Software: Deeptools, GNU parallel, tabix
- Python package: umap-learn, leidenalg, igraph
- R package: reticulate, leiden, data.table, Matrix, irlba, Rtsne, RANN, igraph, uwot, rGREAT, ggplot2

**Data Availability**

We applied CUT&RUNTools 2.0 to a single-cell CUT&Tag dataset available on SRA database (SRP190015 and SRP175327). For the purpose of comparison between bulk and single-cell data, raw FASTQ data of H3K27me3 bulk CUT&Tag for H1 (SRX5193360) and K562 (SRX5193370) were downloaded and processed using CUT&RUNTools 2.0 bulk-data analysis pipeline. The RNA-seq data for H1 (ENCBS734AAA) and K562 (ENCBS864OKZ) were downloaded from the ENCODE website. The genomic annotation data were downloaded from UCSC table browser. The CUT&RUNTools 2.0 software is freely available under the MIT license.

**References:**

1. Langmead,B. and Salzberg,S.L. (2012) Fast gapped-read alignment with Bowtie 2. *Nat. Methods*, **9**, 357–359.

2. Li,H., Handsaker,B., Wysoker,A., Fennell,T., Ruan,J., Homer,N., Marth,G., Abecasis,G. and Durbin,R. (2009) The Sequence Alignment/Map format and SAMtools. *Bioinformatics*, **25**, 2078–2079.

3. Quinlan,A.R. and Hall,I.M. (2010) BEDTools: A flexible suite of utilities for comparing genomic features. *Bioinformatics*, **26**, 841–842.

4. Tange,O. Gnu parallel-the command-line power tool.

5. Zhang,Y., Liu,T., Meyer,C.A., Eeckhoute,J., Johnson,D.S., Bernstein,B.E., Nussbaum,C., Myers,R.M., Brown,M., Li,W., *et al.* (2008) Model-based analysis of ChIP-Seq (MACS). *Genome Biol.*, **9**.

6. Starmer,J. and Magnuson,T. (2016) Detecting broad domains and narrow peaks in ChIP-seq data with hiddenDomains. *BMC Bioinformatics*, **17**.

7. Meers,M.P., Tenenbaum,D. and Henikoff,S. (2019) Peak calling by Sparse Enrichment Analysis for CUT&RUN chromatin profiling. *Epigenetics and Chromatin*, **12**.

8. Amemiya,H.M., Kundaje,A. and Boyle,A.P. (2019) The ENCODE Blacklist: Identification of Problematic Regions of the Genome. *Sci. Rep.*, **9**.

9. Skene,P.J., Henikoff,J.G. and Henikoff,S. (2018) Targeted in situ genome-wide profiling with high efficiency for low cell numbers. *Nat. Protoc.*, **13**, 1006–1019.

10. http://matrix.r-forge.r-project.org/.

11. Dumais,S.T. (2004) Latent Semantic Analysis. *Annu. Rev. Inf. Sci. Technol.*, **38**, 188–230.

12. Traag,V.A., Waltman,L. and van Eck,N.J. (2019) From Louvain to Leiden: guaranteeing well-connected communities. *Sci. Rep.*, **9**.

13. Csárdi,G. and Nepusz,T. The igraph software package for complex network research.

14. Ramírez,F., Dündar,F., Diehl,S., Grüning,B.A. and Manke,T. (2014) DeepTools: A flexible platform for exploring deep-sequencing data. *Nucleic Acids Res.*, **42**, W187.

15. Moudgil,A., Li,D., Hsu,S., Purushotham,D., Wang,T. and Mitra,R.D. (2020) The qBED track: a novel genome browser visualization for point processes. *bioRxiv*, 10.1101/2020.04.27.060061.

16. Li,D., Hsu,S., Purushotham,D., Sears,R.L. and Wang,T. (2019) WashU Epigenome Browser update 2019. *Nucleic Acids Res.*, **47**, W158–W165.

17. Pinello,L., Farouni,R. and Yuan,G.C. (2018) Haystack: Systematic analysis of the variation of epigenetic states and cell-type specific regulatory elements. *Bioinformatics*, **34**, 1930–1933.

18. McLean,C.Y., Bristor,D., Hiller,M., Clarke,S.L., Schaar,B.T., Lowe,C.B., Wenger,A.M. and Bejerano,G. (2010) GREAT improves functional interpretation of cis-regulatory regions. *Nat. Biotechnol.*, **28**, 495–501.

**
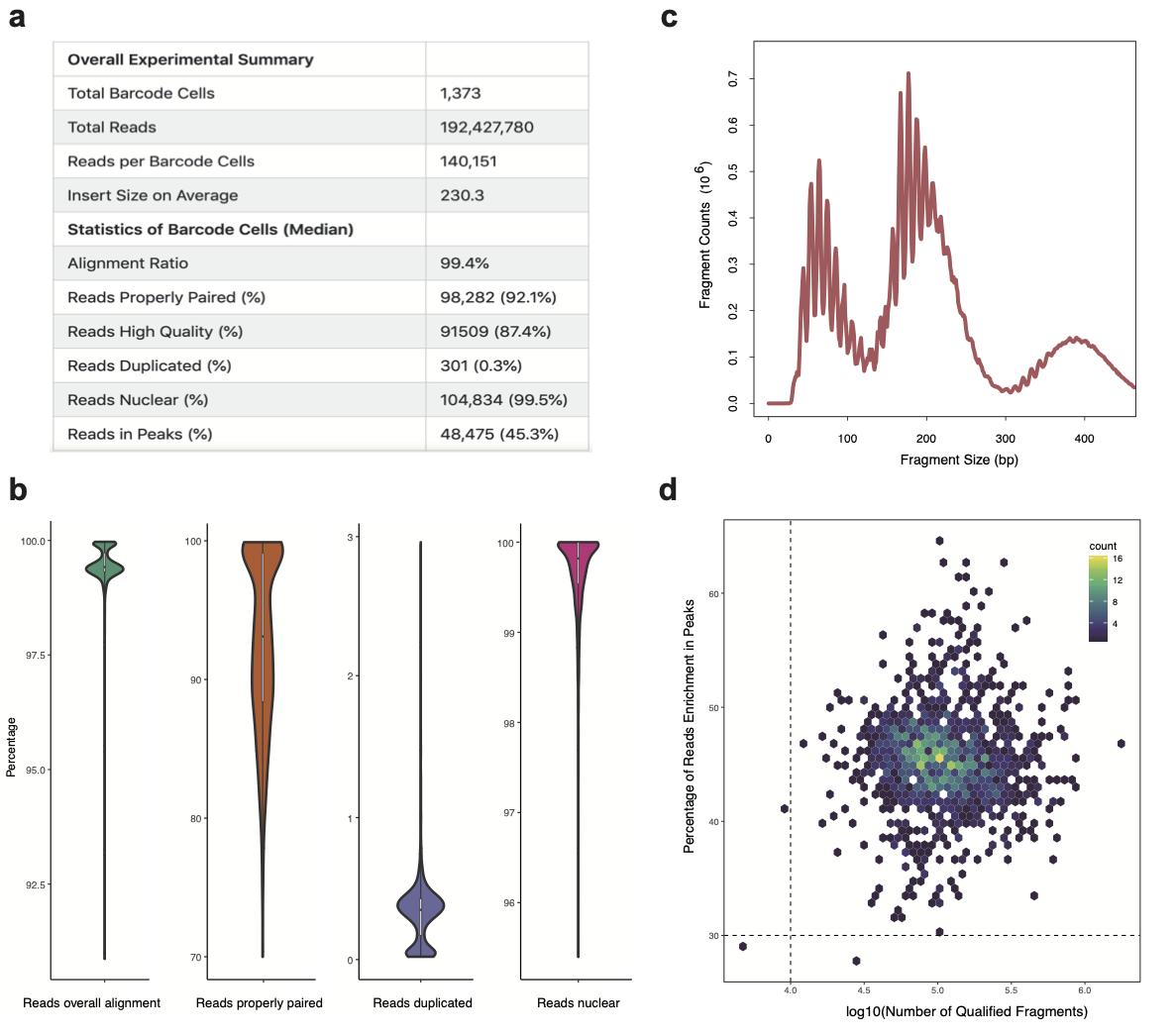
**

**Fig. S1.** **Quality evaluation of single-cell CUT&Tag data.** (a) The overall statistics of the experiment are shown in the summary report. (b) Violin plots for percentage statistics of overall alignment, properly paired reads, duplication reads and nuclear reads. (c) Distribution of fragment size of the qualified fragments for all the cells. (d) Scatter plot of the fraction of unique fragments in peaks versus the total number of unique fragments for each cell. The default thresholds for the two parameters were indicated as dash lines.

**
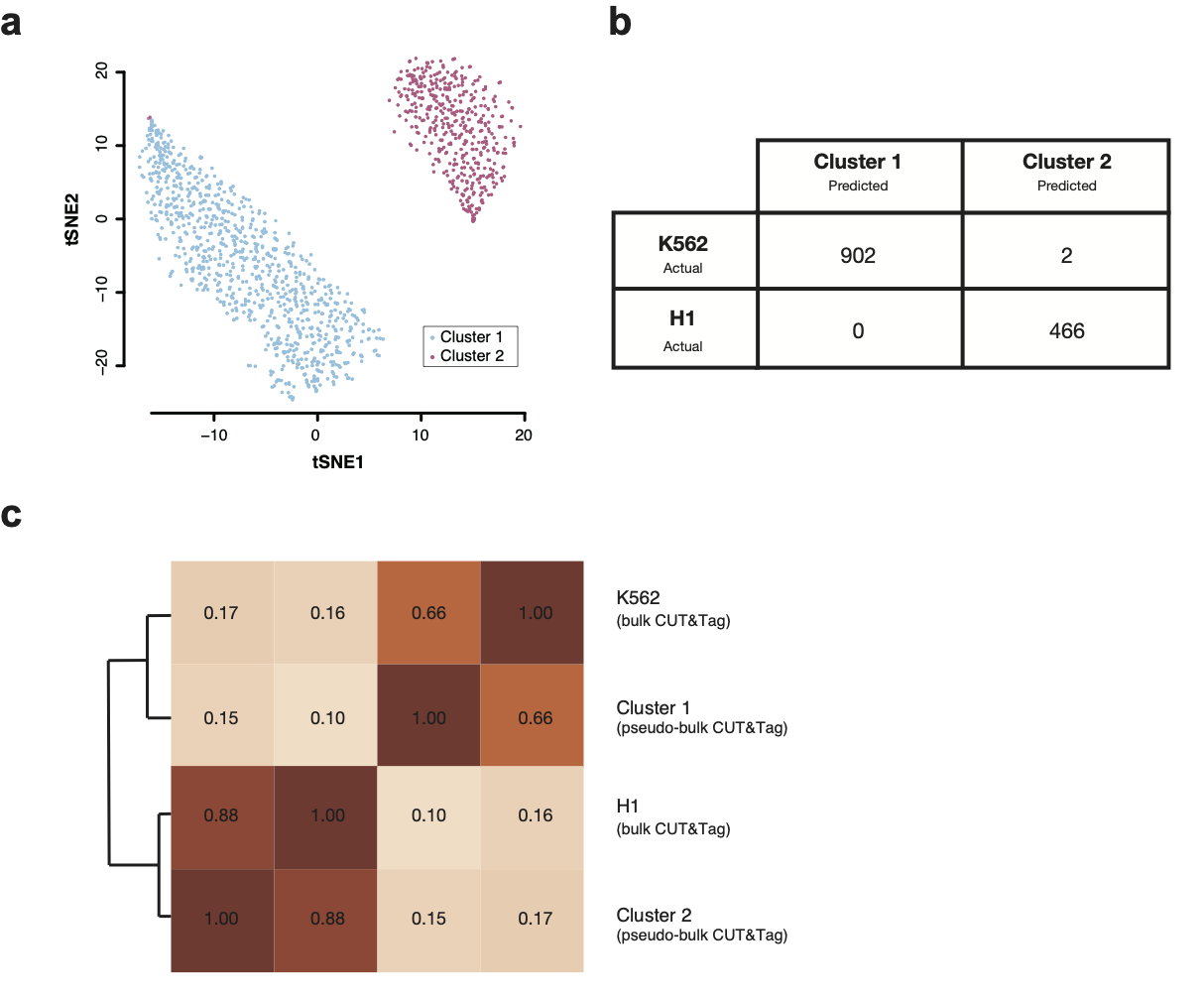
**

**Fig. S2. Analysis of single-cell CUT&Tag data identifies two cell clusters.** (a) A plot of two-dimensional projection of the single-cell CUT&Tag data using the t-SNE method. (b) Confusion matrix of the ground truth cell labels and cell clusters predicted by CUT&RUNTools 2.0. (c) The genome-wide bins with 1 kb resolution were generated and the fragment within each bin was counted for the bulk and pseudo-bulk data of H1 and K562, respectively. The pair-wise Pearson correlation coefficients were calculated and shown in the heatmap.

**
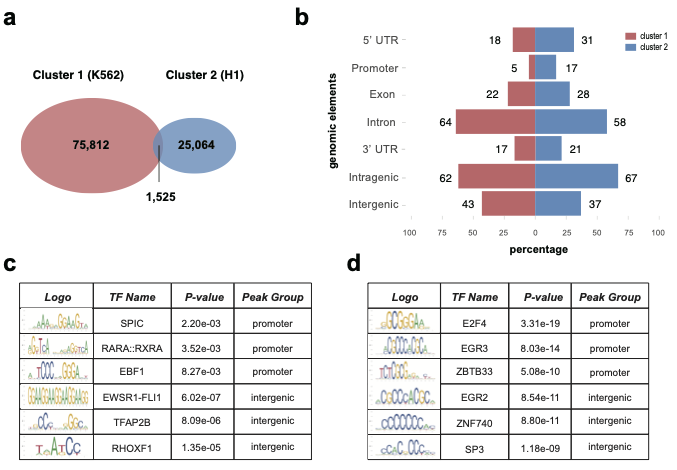
**

**Fig. S3. Functional analysis of the pseudo-bulk data**. (a) Venn diagram illustrates the number of H3K27me3 modification peaks detected in the pseudo-bulk data corresponding to the two cell populations. (b) The percentage distribution of top 5000 peaks overlapping with annotated genomic elements. (c-d) The top enriched TFs associated with different genomic elements of cell cluster1 (c) and cell cluster 2 (d).
